# Enlargement of the Tonsils—I

**Published:** 1908-08-29

**Authors:** 


### Laryngology and Rhinology.

### ENLARGEMENT OF THE TONSILS—I.

Chronic swelling of the tonsils is an affection of childhood and early adult life; it rarely originates after the twenty-fifth year, though, having begun earlier, it may persist to any age in spite of the normal tendency to atrophy shortly after adolescence.

Tonsillar enlargement is in general due to the same causes as adenoid hypertrophy, with which, especially in young children, it is nearly always associated. These causes are not as yet very definitely understood; lymphoid tissue is very active in childhood, and readily responds by hypertrophy to any irritation. When this is markedly the case the child is said to be of the "strumous diathesis," but it does not help the explanation further to say that the strumous diathesis is the cause of enlarged tonsils and adenoids. Heredity appears to have an influence, for when several children in one family are affected the parents often show signs of having had the same trouble themselves in their childhood. Climate plays an important part, and the affection is much commoner in England than in warm, dry countries. The trouble frequently dates from an attack of the exanthemata, such as measles, diphtheria, or scarlet fever.

### The Two Types.

Two types of chronic enlargement of the tonsils are to be observed, according as the lymphoid tissue or the tonsillar crypts are chiefly affected. The former class, which may be called "chronic parenchymatous" swelling, occurs chiefly in children, and is generally associated with adenoids; the tonsil is round and smooth, the epithelium over it seems healthy, and there is little appearance of inflammation; the symptoms present are mostly those due to the size of the enlargement and to the associated adenoids.

In the other type, which affects the crypts, and which may be named "chronic lacunar or follicular tonsillitis," the tonsil is less rounded, and is often flattened and irregular or partially buried beneath an adherent anterior pillar; the surface is pitted by the dilated orifices of the tonsillar crypts, which may contain yellow cheesy masses of inspissated secretion. In this form symptoms of chronic irritation and of toxic absorption are more common, and it is generally found at a somewhat later period of life than the simple parenchymatous swelling. In a later stage, as is seen in older people, fibrosis has occurred, but even now secretions may collect and decompose in the irregular hollows, or the tonsil may be subject to recurring inflammation; it is in this class of case that severe hæmorrhage is most to be feared after excision.

### Symptoms.

The only complaint may be of recurring attacks of acute or subacute tonsillitis, and in the intervals there may be complete absence of symptoms. Or the symptoms may be caused mechanically by the size of the enlargement; such symptoms are more often due to the coexistence of adenoids, but there is no doubt that respiration and even deglutition may be markedly interfered with by great enlargement of the tonsils. An enlarged tonsil, also, may interfere mechanically with the movements of the soft palate, and so with the ventilation of the Eustachian tube and thereby with the middle ear.

Other symptoms of chronic enlargement are those of irritation and discomfort in the throat, pain shooting to the ears, and reflex cough. Finally, there is an important group of symptoms due to the absorption of toxins and of micro-organisms. Secretions accumulate and decompose in the diseased tonsillar crypts, and, when swallowed, produce various digestive disturbances, anæmia and general debility. But more important than this are the effects of the entrance of living micro-organisms through the tonsils into the lymphatic vessels and glands. In the majority of cases of cervical adenitis, the common "glands in the neck," the infecting organisms have gained admission through the faucial ring of lymphoid tissue, and especially through the tonsils.

### A Portal for the Tubercle Bacillus.

As cervical adenitis is generally tuberculous in nature it appears that the tonsils are an important portal for the entry of the tubercle bacillus, and further, it is at least probable that in many cases of pulmonary phthisis the pathway of infection is through the cervical lymphatics to the apex of the lung. The tubercle bacillus can pass through the mucous membrane both of the tonsil and of the intestine into the lymphatic vessels without producing any lesion at the site of entry, and, indeed, the tonsils are very rarely clinically tuberculous. It may be argued that the function of the tonsil is to arrest and destroy bacilli; but, when diseased, it certainly does not do this effectively, and it is a fact of clinical experience that enlarged glands in the neck frequently subside after the removal of unhealthy tonsils.

Indeed, the protective function of the tonsil is probably somewhat overestimated, as the following considerations tend to show. Thus the tonsils normally atrophy in early adult life although the need for protection apparently remains as great as ever; infection of the cervical lymphatic glands occurs almost entirely in children, at a period when the faucial lymphoid tissue is most developed; and that part of the lymphoid ring which is usually the most developed is situated in the naso-pharynx and is the least exposed to micro-organisms.

Besides the tubercle bacillus, many other pathogenic micro-organisms gain admission into or through the tonsils. Diphtheria is usually a primary infection of the tonsil, and it is possible that scarlet fever is also. The organism of rheumatism doubtless often enters by this route, and so may those of various septic diseases, including osteo-myelitis.

